# Extracellular hemoglobin - mediator of inflammation and cell death in the choroid plexus following preterm intraventricular hemorrhage

**DOI:** 10.1186/s12974-014-0200-9

**Published:** 2014-12-02

**Authors:** Magnus Gram, Snjolaug Sveinsdottir, Magnus Cinthio, Kristbjörg Sveinsdottir, Stefan R Hansson, Matthias Mörgelin, Bo Åkerström, David Ley

**Affiliations:** Department of Pediatrics, Lund University, Lund, S-221 84 Sweden; Division of Infection Medicine, Lund University, Lund, S-221 84 Sweden; Department of Electrical Measurements, Lund University, Lund, S-221 84 Sweden; Department of Obstetrics and Gynaecology, Lund University, Lund, S-221 84 Sweden

**Keywords:** Intraventricular hemorrhage, Post-hemorrhagic hydrocephalus, Preterm birth, Choroid plexus, Epithelial cells, Inflammation, Tissue damage, Cell death, Hemoglobin, Haptoglobin

## Abstract

**Background:**

Intraventricular hemorrhage (IVH) with post-hemorrhagic ventricular dilatation (PHVD) is a major cause of neurodevelopmental impairment and mortality in preterm infants. The mechanisms leading to PHVD and brain damage remain largely unknown. The choroid plexus and the ependyma, which constitute an essential part of the blood-brain barrier (BBB), are the first structures to encounter the damaging effects of extravasated blood. The breakdown of the BBB is a critical upstream event leading to brain damage following IVH. In this study we investigated the impact of hemorrhage and hemoglobin (Hb) metabolites on the choroid plexus epithelium.

**Methods:**

Using a preterm rabbit pup model of IVH, the structural and functional integrity, cellular, inflammatory and oxidative response of the choroid plexus, at 24 and 72 hours following IVH + PHVD, were investigated. In order to further characterize cellular and molecular mechanisms, primary human choroid plexus epithelial cells were exposed to cerebrospinal fluid (CSF) from preterm infants with IVH as well as to Hb-metabolites. Finally, the blocking effects of the Hb-scavenger haptoglobin (Hp) were investigated both *in vivo* and *in vitro*.

**Results:**

Following IVH + PHVD, an up-regulation of mRNA for the receptor-related genes TLR-4, IL1R1, FAS, the transcription factor NF-Κβ and for the pro-inflammatory and chemotactic effector molecules, IL-1β, TNFα, MCP-1, IL-8, and IL-6 was observed in the choroid plexus at 24 and 72 hours. This was associated with structural disintegration, caspase activation and cell death in the choroid plexus epithelium. *In vitro* characterization of choroid plexus epithelial cells, following exposure to hemorrhagic CSF and to the Hb-metabolites metHb and heme, displayed apoptotic and necrotic cell death and an up-regulation of receptor-related and inflammatory effector molecules similar to that observed *in vivo* following IVH + PHVD. Intraventricular injection of the Hb-scavenger Hp *in vivo* and co-incubation with Hp *in vitro* reversed or reduced the cellular activation, inflammatory response, structural damage and cell death*.*

**Conclusion:**

Hb-metabolites are important causal initiators of cell death following IVH and removal or scavenging of Hb-metabolites may present an efficient means to reduce the damage to the immature brain following IVH.

## Background

Intraventricular hemorrhage (IVH) is the most common brain lesion in preterm infants with 15 to 20% of very preterm infants developing severe IVH [1,2]. The mortality of infants with severe IVH is 20 to 50% in the neonatal period and of surviving infants approximately 50% develop post-hemorrhagic ventricular dilatation (PHVD) [3-6] and 40 to 80% suffer from disabilities such as cerebral palsy and mental retardation [3-5,7].

Following IVH, the cellular and molecular mechanisms causing brain injury and PHVD are incompletely understood. Post-hemorrhagic hydrocephalus has been explained by mechanistic models, such as clogging of the cerebral aqueduct or the interventricular foramina by coagulated blood leading to an accumulation of cerebrospinal fluid (CSF) as well as impaired CSF absorption [8].

The choroid plexus, which is a collection of villi composed of a single layer of highly specialized ependymal cells overlying a core of intercellular matrix and fenestrated capillaries, produces the CSF. In preterm infants with IVH, rupture of the germinal matrix vasculature leads to the deposition of extravasated blood in the intraventricular space which further hemolyses releasing extracellular hemoglobin (Hb) into the CSF [9]. It is widely recognized in the context of adult intracranial hemorrhage (ICH) that blood and cell-free Hb activate cytotoxic, oxidative and inflammatory pathways leading to tissue damage and cell death [10,11]. Furthermore, studies have shown that metabolites of extracellular Hb have pro-inflammatory effects in microglia, endothelial cells, and macrophages and indeed may behave as activators of innate immunity [12-14] and we recently showed that accumulated levels of metHb (with oxidized, ferric (Fe^3+^) iron as opposed to ferrous (Fe^2+^) iron) in the CSF were highly correlated to TNFα levels in intraventricular CSF at 72 hours following preterm IVH [9].

Following IVH, the choroid plexus is directly exposed to the extravasated blood. However, to our knowledge the effects on the choroid plexus ependymal integrity and function have not been studied. The breakdown of the blood-brain barrier (BBB) is a key event in the development of brain damage following IVH and understanding the molecular mechanisms leading to choroid plexus malfunction will have implications regarding the BBB integrity.

Here, we hypothesize that IVH leads to structural and functional damage to the choroid plexus and that extracellular Hb and its breakdown products are causal initiators of cellular activation, inflammatory response, oxidative stress and subsequent tissue damage and cell death. This was investigated *in vivo* using a preterm rabbit pup model of IVH [15,16] and *in vitro* in primary human choroid plexus epithelial cells. The preterm rabbit pup model is well suited for the study of molecular mechanisms and events of preterm IVH [16-19] since preterm rabbit pups have a germinal matrix and develop spontaneous IVH similar to that of human infants. Furthermore, they exhibit a brain maturation corresponding to that of a human infant at 28 to 30 weeks gestation [18]. The hemorrhage is confined to the intraventricular space and results in a progressive ventricular enlargement very similar to that seen in preterm human infants [19]. Following IVH + PHVD there was a significant structural disintegration of the choroid plexus epithelium accompanied by a distinct cellular activation and an up-regulation of inflammatory mediators and oxidative stress in the choroid plexus at 24 and 72 hours. Furthermore, *in vitro* characterization of choroid plexus epithelial cells, following exposure to hemorrhagic CSF and the Hb-metabolites metHb and heme, revealed increased cell death, cellular activation, inflammatory response and oxidative stress. Finally, co-incubation (*in vitro*) or intraventricular injections (*in vivo*) of the Hb-scavenger haptoglobin (Hp) significantly reversed or reduced the structural damage, inflammatory response, cellular activation and oxidative stress.

## Methods

### Animals

The experiments were performed on a total of 42 rabbit pups from 10 litters delivered at gestational day 29 (term 32 days). A half-breed between New Zealand White and Lop was used. The pups were delivered by caesarean section after the does were anesthetized with intravenous (iv) propofol (5 mg/kg) and with local infiltration of the abdominal wall using lidocaine with adrenaline (10 mg/ml +5 μl/ml, 20 to 30 ml). After birth the pups were dried vigorously, weighed and placed in an infant incubator with a constant temperature of 36°C and 60% ambient humidity. At 2 to 3 hours of age the pups were hand-fed with 1 ml of cat milk formula (KMR; PETAG Inc, Hampshire, IL, USA) using 3.5 French feeding tube, thereafter every 12 hours, increasing each meal by 0.5 ml. At 2 hours of age, the pups received an intraperitoneal (ip) injection of 50% glycerol (6.5 g/kg) to induce intracerebral hypotension due to hyperosmolality, thus causing rupture of the small vessels in the germinal matrix. Ultrasound imaging of the brain was performed at 6 hours of age to detect and grade IVH and after that at 24, 48 and 72 hours of age using the VisualSonics Vevo 2100 (VisualSonics Inc., Toronto, ON, Canada) with a MS-550D 40 MHz transducer. Only animals with a large IVH at six hours were used for data analysis and animals with no detectable IVH at all time-points on cranial ultrasound were used as controls. Measurements of ventricular size for assessment of PHVD were obtained at the level of the midseptal nucleus in a coronal view at 6, 24, 48 and 72 hours of age. Each ventricle was measured horizontally from the mid-brain plane to the lateral wall of the ventricle. Reproducibility and accuracy of ventricular measurements in this animal model using high-frequency ultrasound have been described previously [19]. The animals were euthanized at either 24 hours or 72 hours by an intracardiac injection of pentothal (100 mg/ml, 0.5 ml) and after that the brains were removed from the skull as described below. The animal protocols were approved by the Swedish Animal Ethics Committee in Lund.

### Intraventricular injections

Pups were randomized to receive intraventricular injections of either Hp (Bio Products Laboratory, London, UK) (n = 6) or vehicle solution (Sham, n = 6). After establishing IVH at 6 hours of age the pups received either 20 μl Hp (50 mg/ml) or 20 μl sterile vehicle solution using a 27G Hamilton syringe. For these procedures the rabbit pups were gently fixated on a pre-heated thermostat-controlled platform at 39°C in a prone position with the probe hand-held by one of the investigators and another investigator performed the needle-insertion under ultrasound guidance. The procedure was performed without sedation of the rabbit pups. The efficacy and accuracy of this method has previously been described [19].

### Tissue collection and processing

Rabbit pups were euthanized at 24 (IVH + PHVD, n = 6; sham control n = 6; IVH + Hp, n = 6; IVH + Sham, n = 6) and 72 hours of age (IVH + PHVD n = 9; sham control n = 9), and the brains were removed from the skulls and sectioned at the level of the midseptal nucleus. The choroid plexus was carefully removed from the lateral ventricles, snap frozen, and stored at -80°C until further mRNA and protein analysis as described below. For electron microscopy immunostaining (EM-IHC), the choroid plexus from 2 pups with IVH + PHVD, 2 sham controls, 2 IVH + Hp and 2 IVH + Sham at 24 hours and 2 rabbit pups with IVH + PHVD and 2 sham controls at 72 hours was fixed and prepared as described below. For immunohistochemistry (IHC), the choroid plexus was removed and placed in formaldehyde from 2 pups with IVH + PHVD, 2 sham controls, 2 IVH + Hp and 2 IVH + Sham at 24 hours and in 3 pups with IVH + PHVD and 2 sham controls at 72 hours of age and prepared as described below.

### Cerebrospinal fluid sampling from preterm infants

CSF was sampled from four preterm infants (gestational age at birth 25 to 28 weeks) at 6 to 11 days after detection of IVH, by spinal tap or ventricular reservoir puncture according to clinical routine in the neonatal unit at Lund University Hospital. Immediately after sampling, the CSF was centrifuged (2,000 × g, 20°C for 10 minutes), pooled and Hb- and Hb-metabolite (that is oxyHb and metHb) concentrations were determined in CSF from preterm infants using Plasma/Low Hb (Hemocue, Ängelholm, Sweden) and a spectrophotometric method described previously [20]. Samples were stored at -80°C until further use, as described below. The sampling was performed following written consent from the parents, and the study was approved by the ethical committee review board for studies in human subjects at Lund University.

### Preparation of methemoglobin and heme

Fetal Hb was purified as previously described [21] from freshly drawn human umbilical cord blood. MetHb was prepared by incubating the purified Hb solution at 37°C for 72 hours. The metHb concentration was quantified as described previously [21]. Heme (ferriprotoporphyrin IX chloride) was purchased from Porphyrin Products Inc. (Logan, UT, USA), and a 10 mM stock solution was prepared using dimethyl sulfoxide (DMSO; Sigma-Aldrich, St. Louis, MO, USA). The metHb was purified from endotoxin contamination using the endotoxin removing product EndoTrap (Hyglos GmbH,Bernried am Starnberger See, Germany) as described by the manufacturer. The absolute purity of metHb and heme from contamination with endotoxin (0 EU/mg Hb/heme) was determined using the QCL-1000™ Endpoint Chromogenic LAL Assay (Lonza, Switzerland) as described by the manufacturer.

### Primary human choroid plexus epithelial cells

Human primary choroid plexus epithelial cells (HCPEpiC, ScienCell, Carlsbad, CA, USA) were cultured in epithelial cell medium containing 2% fetal bovine serum, 1% epithelial cell growth supplement, 100 units/ml penicillin and 100 μg/ml streptomycin (all ScienCell, Carlsbad, CA, USA). When cells reached approximately 90% confluence pooled CSF from 4 preterm infants with IVH, metHb and heme (prepared immediately prior to the experiment, as described above) were added to the HCPEpiC cultures, and cells were incubated for 1 to 24 hours as indicated in the figure legends. After incubation, cells were harvested using either Qiazol™ lysis reagent (for RNA extraction, QIAGEN, Germantown, MD, USA) or cell extraction buffer (for protein extraction, Invitrogen, Camarillo, CA, USA). Total RNA and protein was extracted from cells to evaluate mRNA expression and protein content, as described below.

### RNA isolation and real-time PCR

Total RNA was isolated from choroid plexus and HCPEpiC cells using the acid guanidinium phenol chloroform method and RNeasy Mini Kit supplied by QIAGEN (Germantown, MD, USA). The optical density (OD) ratio (OD at 260 nm/280 nm) of RNA was always higher than 1.9. Reverse transcription was performed according to manufacturer on 0.1 to 1 μg total RNA using iScript™ cDNA Synthesis Kit (Bio-Rad, Hercules, CA, USA) and RT^2^ First Strand Kit (QIAGEN, Germantown, MD, USA). RT^2^ PCR Profiler Array real-time PCR (custom made by QIAGEN, Germantown, MD, USA) were used to quantify the mRNA expression of TLR-4, IL1R1, FAS, NF-Κβ, MCP-1, IL-8, IL-1β, TNFα, IL-6 and HO-1. Data were normalized to glyceraldehyde-3-phosphate dehydrogenase (GAPDH, custom made by QIAGEN; Germantown, MD, USA). The fold change values were calculated by normalizing against control samples from untreated animals or cells. Data are presented as box plots, displaying medians and 25^th^ and 75^th^ percentiles, for *in vivo* data and as bars, displaying mean ± SEM, for *in vitro* data. Expression was analyzed using RT^2^ SYBR Green Fluor qPCR Mastermix (QIAGEN, Germantown, MD, USA). Amplification was performed as described by the manufacturer (QIAGEN) for 40 cycles in an iCycler Thermal Cycler (Bio-Rad, Hercules, CA, USA) and data analyzed using iCycler iQ Optical System Software (Bio-Rad, Hercules, CA, USA).

### Total protein analysis

Total protein from choroid plexus was determined by Pierce®BCA Protein Assay Kit (Thermo Scientific, Rockford, IL, USA).

### Histology

Tissues were fixed in 4% paraformaldehyde, according to routine protocols. Following paraffin embedding, tissues were sectioned at 3-μm and subsequently stained with H&E as described by the manufacturer (Histolab Products AB, Gothenburg, Sweden).

### Immunohistochemistry

Immunohistochemistry sections were deparaffinized by routine procedures and endogenous peroxidase activity was blocked with 3% H_2_O_2_ in methanol for 15 minutes. After washing with Triton-X-100 (0.25%) in PBS, sections were blocked with normal goat serum (5%) for 1 hour at room temperature (RT). The slides were then incubated with cleaved caspase-3 primary antibody overnight at 4°C. Antibody detection was performed with a standard avidin-biotin complex detection system after which they were developed with 3,3-diaminobenzidine tetrahydrochloride as the chromogenic substrate (Vectastain avidin-biotin complex, Vector Laboratories, Burlingame, CA, USA). Sections were mounted with Pertex (Histolab, Gothenburg, Sweden) and examined and photographed (Olympus BHS photomicrographic system, Hamburg, Germany).

### Transmission electron microscopy (TEM)

For ultrathin sectioning, choroid plexus were fixed for 1 hour at RT and then overnight at 4°C in 2.5% glutaraldehyde in 0.15 M sodium cacodylate, pH 7.4 (cacodylate buffer). Samples were then washed with cacodylate buffer and post-fixed for 1 hour at RT in 1% osmium tetroxide in cacodylate buffer, dehydrated in a graded series of ethanol, and then embedded in Epon 812 (SPI Supplies, West Chester, PA, USA) using acetone as an intermediate solvent. Specimens were sectioned into 50 to 70-nm-thick ultrathin sections on an LKB ultramicrotome. The ultrathin sections were stained with uranyl acetate and lead citrate. Immunolabeling of thin sections after antigen unmasking with sodium metaperiodate [22] with gold-labeled anti-TNFα was performed as described previously [23] with the modification that Aurion-BSA (Aurion, Wageningen, the Netherlands) was used as a blocking agent. Specimens were observed in a JEOL JEM 1230 electron microscope (JEOL, Peabody, MA, USA) operated at 80 kV accelerating voltage. Images were recorded with a Gatan Multiscan 791 CCD camera.

### TNFα ELISA

The concentrations of TNFα in extracted choroid plexus tissue from rabbit pups were determined using the Rabbit TNFα DuoSet ELISA Development kits from R&D Systems (UK) with a minor adjustment of the protocol. Briefly, calibrator rabbit TNFα was 2-fold serial diluted to 3.9 pg/ml.

### Cell viability assay

The levels of lactate dehydrogenase (LDH) in choroid plexus cell culture media were measured using the CytoTox 96® Non-Radioactive Cytotoxicity Assay (Promega, Madison, WI, USA) according to the manufacturer's instructions.

### Caspase-3/7 assay

The levels of activated caspase-3/7 in choroid plexus cells were measured using the caspase-3/7 assay as described by the manufacturer (Promega, Madison, WI, USA).

### Statistics

Comparisons between unrelated groups were performed with the Mann-Whitney *U*-test as appropriate. Comparisons between multiple groups were performed by analysis of variance (ANOVA) with *post hoc* Bonferroni correction. *P*-values < 0.05 were considered significant.

## Results

### Structural damage and cell death in the choroid plexus following IVH

**Figure 1:**
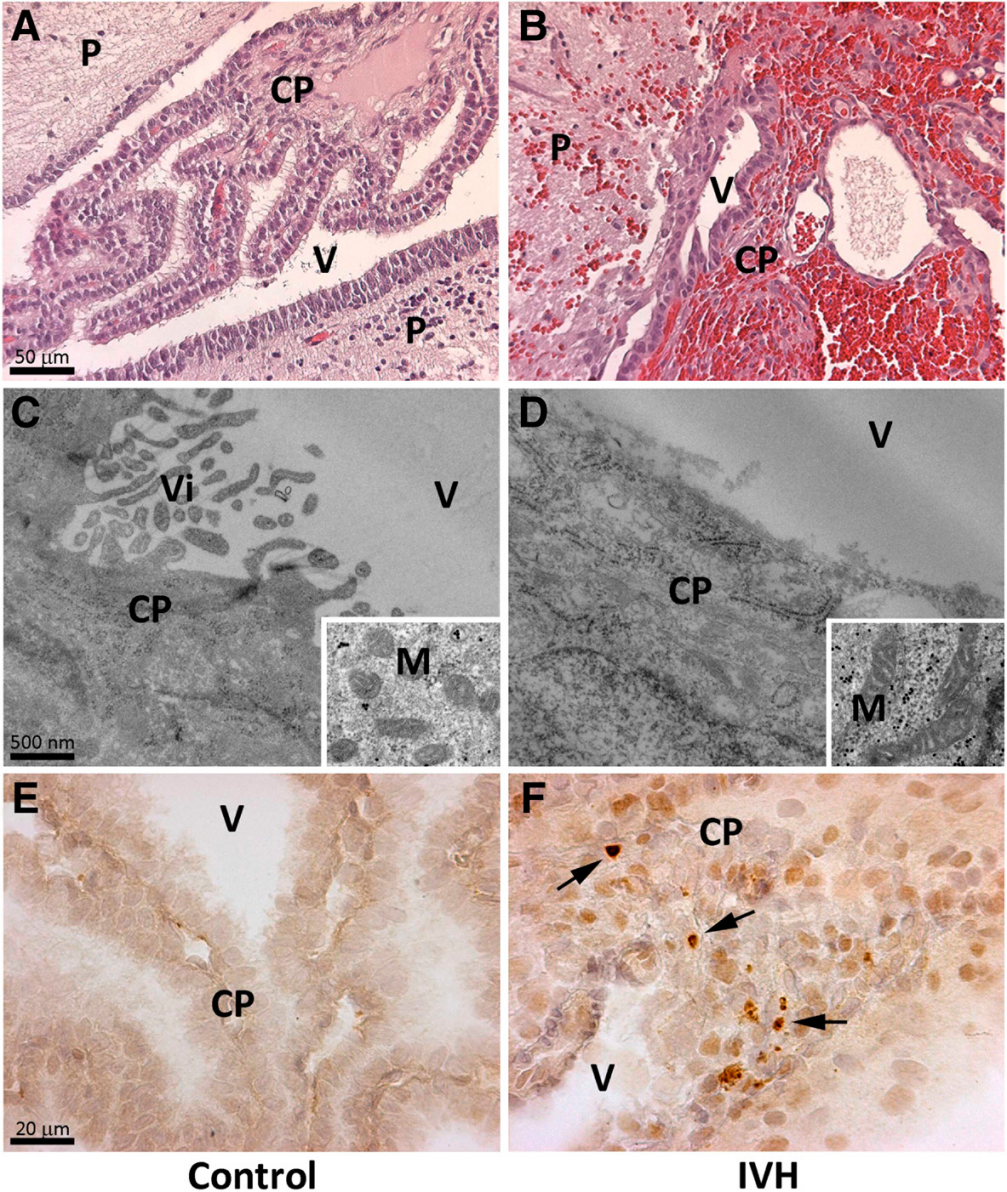
**Analysis of structural damage and cell death in the choroid plexus.** Rabbit pups with IVH + PHVD **(B**, **D**, **F)** or sham controls **(A**, **C**, **E)** were euthanized at 72 hours of age and the brains were removed from the skulls, sectioned at the level of the midseptal nucleus and placed in formaldehyde for subsequent histochemical analysis with H&E **(A** and **B)** and IHC analysis against cleaved caspase-3 **(E** and **F)** as described in Materials and Methods. For EM-IHC **(C** and **D)**, the choroid plexus from pups with IVH + PHVD and sham controls at 72 hours was fixed and prepared as described in the Materials and Methods and observed in a Jeol JEM 1230 electron microscope. Recessed picture display mitochondrial structure. P = periventricular tissue, CP = choroid plexus, V = ventricle, Vi = villi, M = mitochondria. Arrow illustrates cleaved caspase-3 positive staining. Scale bar in **(A)** indicate 50 μm (is applicable for panels **(A)** and **(B)**), in **(C)** indicate 500 nm (is applicable for **(C)** and **(D)**) and **(E)** indicate 20 μm (is applicable for **(E)** and **(F)**). IHC, immunohistochemistry; IVH, intraventricular hemorrhage; PHVD, post-hemorrhagic ventricular dilatation.

Structural and cellular damage to the choroid plexus was investigated following IVH in preterm rabbit pups. Histopathological analysis with H&E staining displayed extensive structural damage to the epithelial cells at 72 hours after IVH (Figure 1A and B). Substantial ultrastructural damage to the choroid plexus was seen as determined by EM; for example, disintegration of normal epithelial structure and swollen mitochondria (Figure 1C and D). IHC analysis of apoptosis, using antibodies towards cleaved caspase-3, displayed a strong staining at 24 hours following IVH (Figure 1F), which was not observed in the control animals (Figure 1E).

### Cellular activation, inflammatory response and oxidative stress in the choroid plexus following IVH

**Figure 2:**
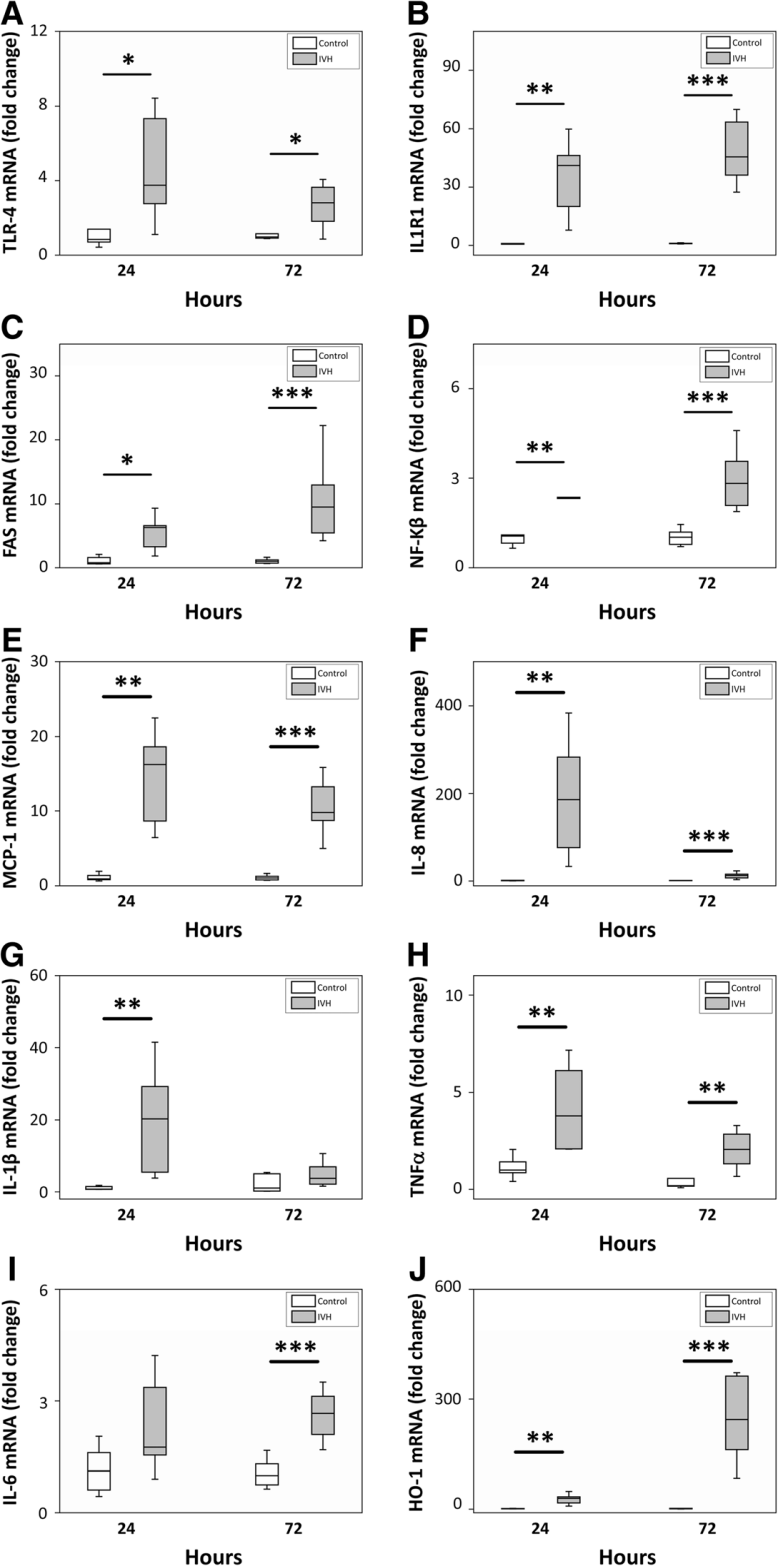
**mRNA expression of cellular activation, inflammatory response and oxidative stress in choroid plexus.** Rabbit pups with IVH + PHVD (grey bars; n = 6 at 24 hours and n = 9 at 72 hours) or sham controls (white bars; n = 6 at 24 hours and n = 9 at 72 hours) were euthanized at 24 and 72 hours of age and the brains were removed from the skulls and the choroid plexus was carefully removed from the lateral ventricles, snap frozen, and the mRNA expression of TLR-4 **(A)**, IL1R1 **(B)**, FAS **(C)**, NF-Κβ **(D)**, MCP-1 **(E)**, IL-8 **(F)**, IL-1β **(G)**, TNFα **(H)**, IL-6 **(I)**, and HO-1 **(J)** were subsequently analyzed with real-time PCR, as described in Materials and Methods. mRNA expression of TLR-4, IL1R1, FAS, NF-Κβ, MCP-1, IL-8, IL-1β, TNFα, IL-6, and HO-1 were normalized against those of GAPDH and are given as fold change. The fold-change values were calculated by normalizing against samples from control pups. Results are presented as box plots displaying medians and 25^th^ and 75^th^ percentiles. Differences between IVH + PHVD versus control at 24 and 72 hours, respectively, were analyzed using the Mann-Whitney *U*-test. *P < 0.05, **P < 0.01, ***P < 0.001. GAPDH, glyceraldehyde-3-phosphate dehydrogenase; IVH, intraventricular hemorrhage; PHVD, post-hemorrhagic ventricular dilatation.

Analysis of mRNA expression of the receptor-related signaling genes TLR-4, IL1R1, FAS and of the transcription factor NF-Κβ displayed a significant up-regulation at 24 and 72 hours following IVH (Figure 2A-D). All genes, except TLR-4, exhibited an increased expression at 72 hours as compared to 24 hours following IVH.

**Figure 3:**
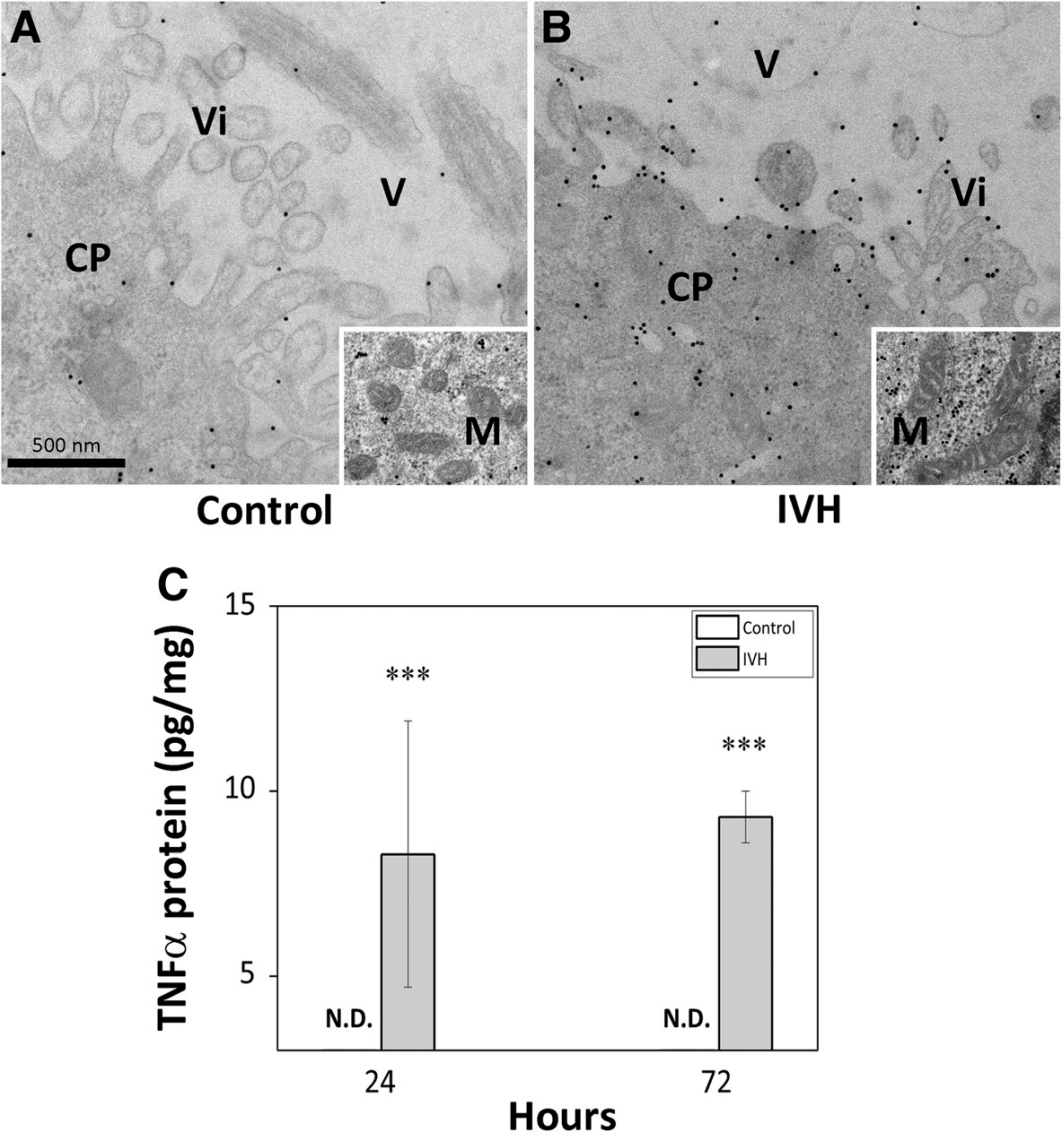
**Analysis of TNFα protein and distribution in choroid plexus.** Rabbit pups with IVH + PHVD **(B and C)** or sham controls **(A and C)** were euthanized at 24 and 72 hours of age and the brains were removed from the skulls. For EM-IHC, the choroid plexus of rabbit pups at 72 hours were fixed and prepared as described in the Materials and Methods. Sections were stained against TNFα and observed in a Jeol JEM 1230 electron microscope. Recessed picture display mitochondrial structure. CP = choroid plexus, V = ventricle, Vi = villi. Scale bar in **(A)** indicate 500 nm (is applicable for panels **(A)** and **(B)**). Analysis of TNFα protein (C) levels in choroid plexus tissue of preterm rabbit pups with IVH + PHVD (grey bars; n = 6 at 24 hours and n = 9 at 72 hours) and in control pups (white bars; n = 6 at 24 hours and n = 9 at 72 hours) using ELISA, as described in the Materials and Methods section. Results were corrected for total protein content and are presented as TNFα/total protein (pg/mg) displaying mean ± SEM. The difference between IVH versus control at 72 hours was analyzed using the Mann-Whitney U-test. ***P < 0.001. IVH, intraventricular hemorrhage; PHVD, post-hemorrhagic ventricular dilatation.

Analysis of inflammatory effector molecule activation revealed a significant up-regulation of MCP-1, IL-8, IL-1β, TNFα and IL-6 following IVH (Figure 2E-I). The expression of MCP-1, IL-8 and TNFα was significantly increased both at 24 and 72 hours, whereas IL-1β was increased at 24 but not at 72 hours and IL-6 was significantly increased at 72 but not at 24 hours. Analysis of mRNA expression of HO-1, a Hb/heme- and oxidative stress-inducible protein responsible for intracellular heme degradation, revealed a highly significant up-regulation at both 24 and 72 hours following IVH (Figure 2J). Analysis of TNFα protein showed an increase at both 24 and 72 hours (Figure 3C). In addition, EM-IHC of TNFα displayed a strong staining in the choroid plexus following IVH at 24 hours (Figure 3A and B) as compared to that in control pups.

### Exposure of choroid plexus cell cultures to hemorrhagic CSF and to Hb-metabolites

**Figure 4:**
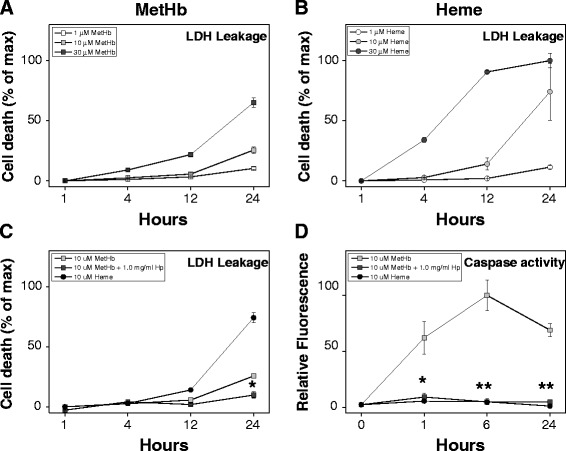
**MetHb- and heme-induced cell death and caspase activity in choroid plexus epithelial cell cultures.** Cell death (leakage of lactate dehydrogenase (LDH)) in HCPEpiC cells, exposed to 1 to 30 μM metHb **(A)**, 1 to 30 μM heme **(B)** or 10 μM metHb with +1.0 mg/ml Hp **(C)**, was evaluated in cell culture medium at 1 to 24 hours as described in Materials and Methods. Caspase activity (activation of caspase-3 and -7) in HCPEpiC following exposure to 10 μM metHb **(□)**, 10 μM heme **(●)** or 10 μM metHb +1.0 mg/ml Hp **(■)** was determined **(D)**. Results are from triplicate experiments and presented as mean ± SEM. Baseline in respective graph corresponds to LDH leakage and caspase activity of control cells (exposed to culture medium only) for respective time point. Statistical differences between 10 μM metHb +1.0 mg/ml Hp versus 10 μM metHb were analyzed using ANOVA *post hoc* Bonferroni. *P < 0.05, **P < 0.01.

The response of choroid plexus to blood and blood components following IVH was further characterized by exposing human primary choroid plexus epithelial cells to CSF from preterm infants with IVH and to isolated Hb-metabolites, metHb and heme. Exposing the plexus cells to hemorrhagic CSF (1 to 30% of cell culture medium volume) showed a small but dose-dependent cell death at 4 hours, whereas exposure to CSF for 24 hours induced a highly significant or almost complete (using 30% CSF) cell death, as measured by LDH-release (not shown). The cell damaging properties of Hb-metabolites (metHb and heme at 1 to 30 μM, corresponding to similar levels of metHb and heme as observed in hemorrhagic CSF) on the plexus epithelial cells was characterized by investigating cell death; for example, necrosis (as determined by LDH leakage) and apoptosis (as determined by caspase-3 and -7 activation) (Figure 4). A dose- and time-dependent cell death was observed for all conditions (Figure 4A and B). Exposure to high concentrations of metHb caused a moderate cell death, reaching a highly significant degree of dead cells following 24 hours exposure to 30 μM metHb (Figure 4A). Exposure to heme caused a substantial amount of dead cells at a moderate concentration (10 μM) and exposure to high concentration (30 μM) caused a highly significant cell death at 12 and 24 hours (Figure 4B). Analysis of apoptosis displayed a strong activation of caspase-3 and -7 following exposure to 10 μM metHb, which was not observed following exposure to 10 μM heme (Figure 4D). As a consequence of these results, 1 to 30% hemorrhagic CSF, 10 μM metHb and 10 μM heme was used in subsequent *in vitro* experiments.

**Figure 5:**
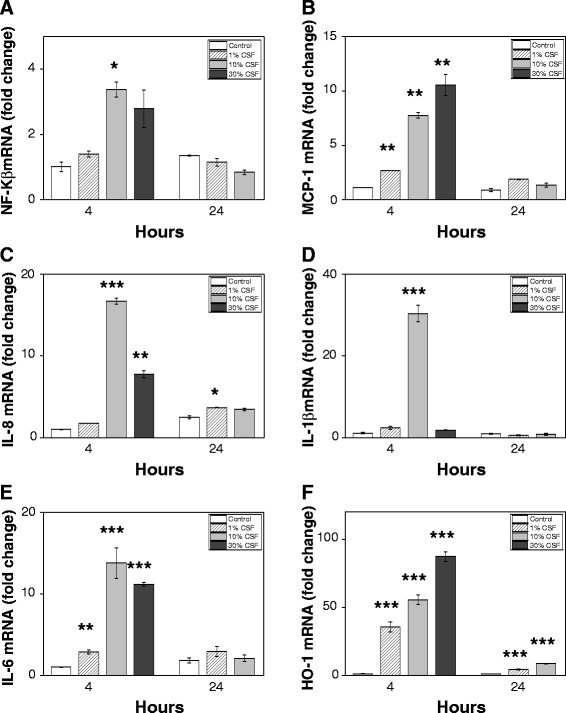
**CSF-induced cellular activation, inflammatory response and oxidative stress in choroid plexus epithelial cell cultures.** mRNA expression of NF-Κβ **(A)**, MCP-1 **(B)**, IL-8 **(C)**, IL-1β **(D)**, IL-6 **(E)** and HO-1 **(F)** in HCPEpiC cells, exposed to culture medium containing 1 to 30% (of cell culture medium volume) CSF from preterm infants with intraventricular hemorrhage (IVH) for 4 and 24 hours was determined using real-time PCR as described in Materials and Methods. The mRNA expression of all respective genes was normalized against glyceraldehyde-3-phosphate dehydrogenase (GAPDH) and is given as fold change. The fold-change values were calculated by normalizing against control samples from untreated cells. Results are from triplicate experiments and presented as mean ± SEM. Differences between the respective exposures versus control for respective time point were analyzed using ANOVA *post hoc* Bonferroni. *P < 0.05, **P < 0.01, ***P < 0.001.

Exposure of choroid plexus epithelial cells to hemorrhagic CSF (1 to 30% of cell culture medium volume) caused a significant increase in NF-Κβ, MCP-1, IL-8, IL-1β and IL-6 mRNA expression at 4 hours (Figure 5A-E). After exposure for 24 hours only a slight increase was observed for MCP-1, IL-8 and IL-6 expression whereas NF-Κβ and IL-1β were slightly down-regulated. Furthermore, in line with the *in vivo* analysis of HO-1 mRNA expression, choroid plexus epithelial cells displayed a dose-dependent up-regulation of HO-1 mRNA both at 4 and 24 hours (Figure 5F). However, the relative levels were significantly higher at 4 hours, most likely as a result of the decreased cell viability observed at 24 hours (not shown).

**Figure 6:**
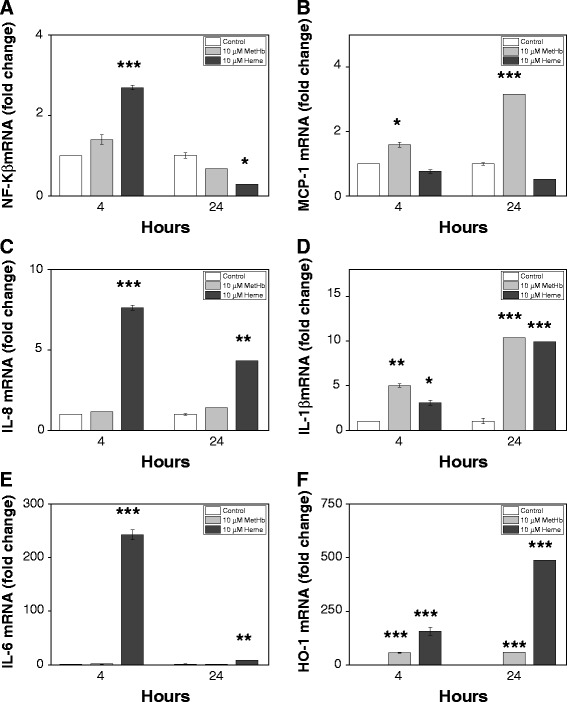
**MetHb- and heme-induced cellular activation, inflammatory response and oxidative stress in choroid plexus epithelial cell cultures.** mRNA expression of NF-Κβ **(A)**, MCP-1 **(B)**, IL-8 **(C)**, IL-1β **(D)**, IL-6 **(E)** and HO-1 **(F)** in HCPEpiC cells, exposed to 10 μM metHb or heme for 4 and 24 hours was determined using real-time PCR as described in Materials and Methods. The mRNA expression of all respective genes was normalized against glyceraldehyde-3-phosphate dehydrogenase (GAPDH) and is given as fold change. The fold-change values were calculated by normalizing against control samples from untreated cells. Results are from triplicate experiments and presented as mean ± SEM. Differences between the respective exposures versus control for respective time point were analyzed using ANOVA *post hoc* Bonferroni. *P < 0.05, **P < 0.01, ***P < 0.001.

Exposing the choroid plexus epithelial cells to the Hb-metabolites metHb and heme resulted in a similar mRNA expression pattern as observed following exposure to hemorrhagic CSF with significant increases in NF-Κβ, MCP-1, IL-8, IL-1β and IL-6 at 4 hours following exposure to 10 μM metHb and/or 10 μM heme (Figure 6A-E). At 24 hours, a significant up-regulation of MCP-1, IL-8, IL-1β and IL-6 was observed. Congruent to the CSF results, exposure to metHb and heme resulted in a down-regulation of NF-Κβ at 24 hours and furthermore analysis of HO-1 mRNA expression displayed a highly significant up-regulation both at 4 and 24 hours following exposure to 10 μM metHb and 10 μM heme respectively (Figure 6F).

Analysis of choroid plexus epithelial cells following exposure to hemorrhagic CSF, metHb or heme displayed no significant mRNA up-regulation of TNFα, IL1R1, TLR-4 and FAS at either 4 or 24 hours (not shown). This is probably due to the more diverse cellular composition of choroid plexus *in vivo*, that is aside from epithelial cells there are also inflammatory cells such as macrophages.

### Scavenging of Hb *in vitro* by Hp inhibits cell death and reduces cellular activation

Scavenging of Hb-metabolites, by simultaneous addition of equivalent molar concentrations of Hp to metHb exposed choroid plexus epithelial cells, showed a highly significant inhibition of cell death (as determined by LDH leakage) and a totally abrogated induction of apoptosis (as determined by caspase-3 and -7 activation) (Figure 4C-D).

**Figure 7:**
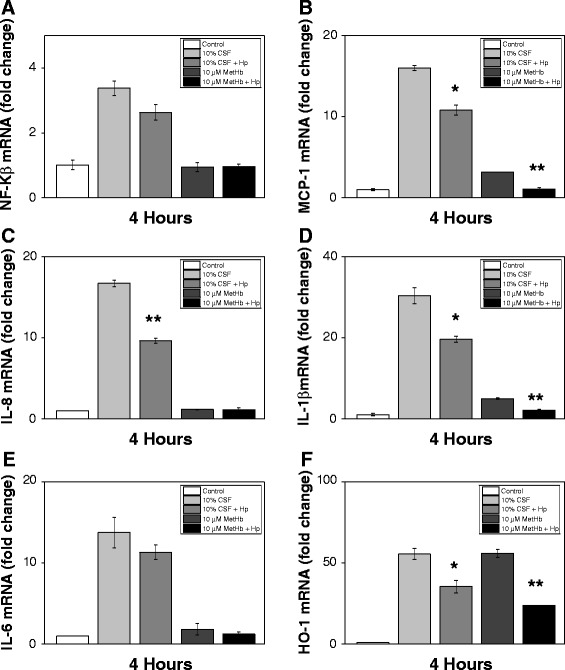
**Hp reduces Hb-induced cellular activation, inflammatory response and oxidative stress in choroid plexus epithelial cell cultures.** mRNA expression of NF-Κβ **(A)**, MCP-1 **(B)**, IL-8 **(C)**, IL-1β **(D)**, IL-6 **(E)** and HO-1 **(F)** in HCPEpiC cells, exposed to 10% (of cell culture medium volume) cerebrospinal fluid (CSF) from preterm infants with IVH or 10 μM metHb with or without the addition of 1.0 mg/ml Hp, for 4 and 24 hours was determined using real-time PCR as described in Materials and Methods. The mRNA expression of all respective genes was normalized against glyceraldehyde-3-phosphate dehydrogenase (GAPDH) and is given as fold change. The fold-change values were calculated by normalizing against control samples from untreated cells. Results are from triplicate experiments and presented as mean ± SEM. Differences between the respective exposures versus control for respective time point were analyzed using ANOVA *post hoc* Bonferroni. *P < 0.05, **P < 0.01.

Furthermore, analysis of the mRNA expression of choroid plexus epithelial cells following exposure to hemorrhagic CSF and metHb with simultaneous Hb-metabolite scavenging by Hp showed a significant reduced expression of NF-Κβ (Figure 7A). Analysis of inflammatory mediators displayed a significantly reduced mRNA expression of MCP-1, IL-8, IL-1β and IL-6 at 4 hours following addition of Hp (Figure 7B-E). Furthermore, analysis of HO-1 mRNA expression, displayed a highly significant reduced expression following Hp addition (Figure 7F). No reduction in TLR-4 expression was observed following co-incubation with Hp.

### Scavenging of Hb *in vivo* by Hp reduces damage, cellular activation, inflammatory response and oxidative stress

**Figure 8:**
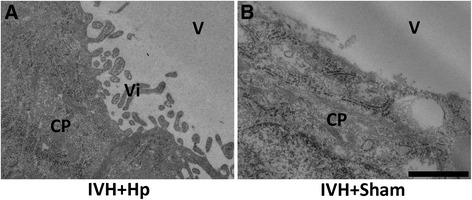
**Hp reduces Hb-induced structural damage and cell death in the choroid plexus.** Rabbit pups with confirmed intraventricular hemorrhage (IVH), injected intraventricularly with Hp **(A**, IVH + Hp) or sham **(B**, IVH + Sham**)** 6 hours after confirmation of the bleeding, were euthanized at 24 hours of age and the brains were removed from the skulls, sectioned at the level of the midseptal nucleus and choroid plexus was fixed and prepared as described in the Materials and Methods and observed in a Jeol JEM 1230 electron microscope. CP = choroid plexus, V = ventricle, Vi = villi. Scale indicates 500 nm.

The importance of Hb-metabolite-induced structural damage, cellular activation, inflammatory response and oxidative stress in the choroid plexus *in vivo* was investigated by an intraventricular injection of the Hb-scavenger Hp or vehicle solution to rabbit pups with confirmed IVH, at 6 hours after confirmation of the bleeding. Examination of the structural integrity of the choroid plexus with EM (Figure 8A-B) at 24 hours after confirmed bleeding displayed a clearly reduced tissue damage following Hp injection, as compared to vehicle solution injection.

**Figure 9:**
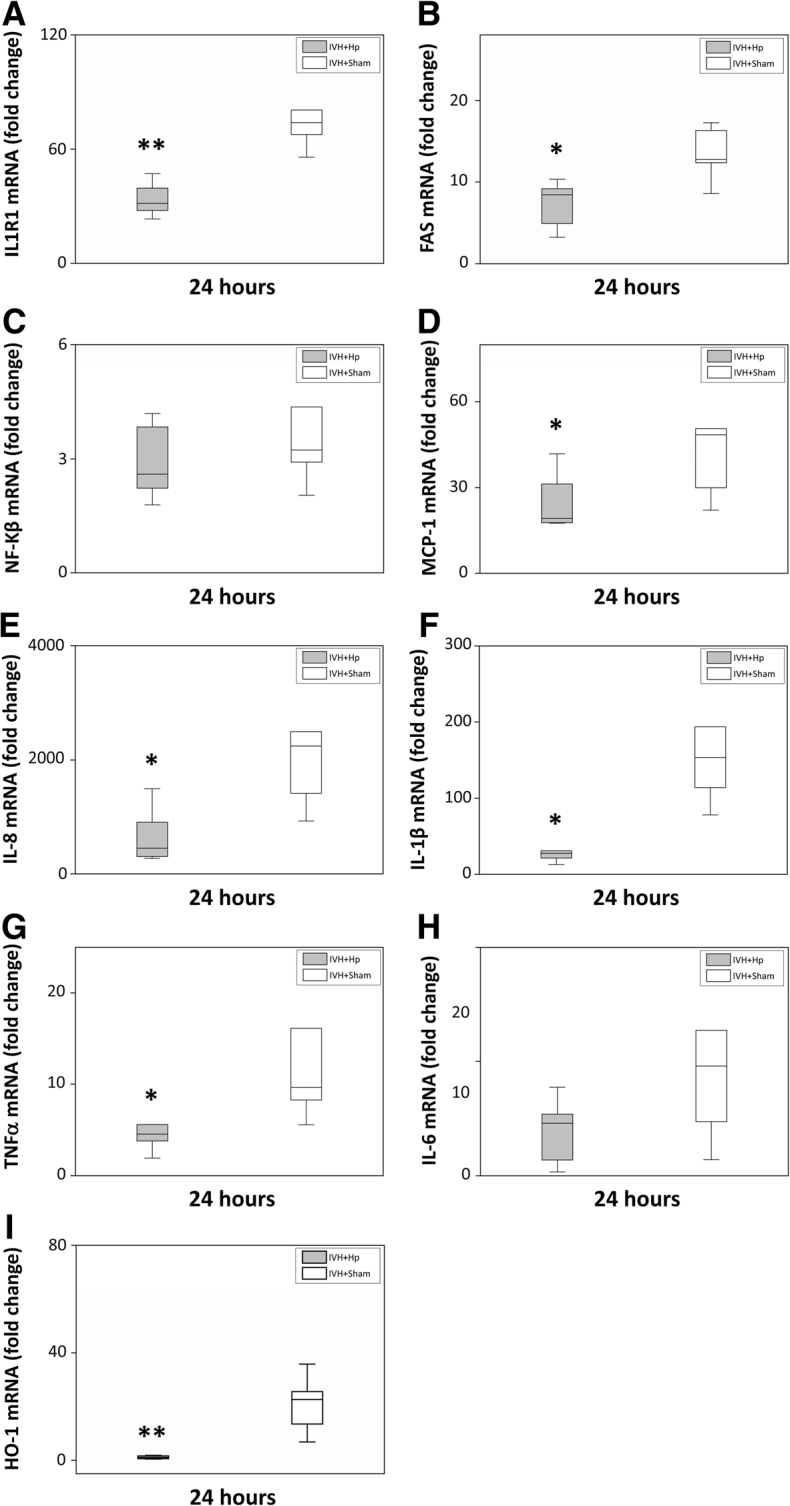
**Hp reduces Hb-induced cellular activation, inflammatory response and oxidative stress in the choroid plexus.** Rabbit pups with confirmed IVH, injected intraventricularly with Hp (grey bars; n = 6) or sham (white bars; n = 6) 6 hours after confirmation of the bleeding, were euthanized at 24 hours of age and the brains were removed from the skulls and the choroid plexus was carefully removed from the lateral ventricles, snap frozen, and the mRNA expression of IL1R1 **(A)**, FAS **(B)**, NF-Κβ **(C)**, MCP-1 **(D)**, IL-8 **(E)**, IL-1β **(F)**, TNFα **(G)**, IL-6 **(H)** and HO-1 **(I)** were subsequently analyzed with real-time PCR, as described in Materials and Methods. mRNA expression for respective gene was normalized against those of glyceraldehyde-3-phosphate dehydrogenase (GAPDH) and is given as fold change. The fold-change values were calculated by normalizing against samples from control pups. Results are presented as box plots displaying medians and 25^th^ and 75^th^ percentiles. Differences between IVH + Hp versus IVH + Sham at 24 hours were analyzed using the Mann-Whitney *U*-test. *P < 0.05, **P < 0.01, ***P < 0.001.

Analysis of cellular activation, inflammatory response and oxidative stress demonstrated a highly significant reduction in IL1R1, FAS, NF-Κβ, MCP-1, IL-8, IL-1β, TNFα, IL-6 and HO-1 mRNA expression following intraventricular injection of Hp, as compared to vehicle solution (Figure 9A-I).

## Discussion

In this study we demonstrate a structural damage to the choroid plexus ependyma 24 hours after IVH, accompanied by an intense cellular activation and inflammatory response. In addition, a severe cellular disintegration with loss of normal villous morphology and signs of cellular apoptosis/necrosis is seen 72 hours after the insult. We show that similar changes are induced in choroid plexus epithelial cells *in vitro* following exposure to hemorrhagic CSF and cell-free Hb-metabolites. Intraventricular injections of the Hb-scavenger Hp *in vivo* and co-incubation with Hp *in vitro* reduced or reversed cellular and inflammatory induction and cell death.

The choroid plexus is a free-floating organ located in the roof of the lateral ventricles and is the first organ to fully encounter the effects of extravasated blood released into the intraventricular space. To our knowledge this study is the first to evaluate the structural damage and molecular response in the choroid plexus following preterm IVH. The choroid plexus plays a vital role in the production and homeostasis of CSF, constitutes an integral part of the BBB but is also an extension of the ependymal lining constituting the CSF to brain barrier. The breakdown of the BBB has indeed been shown to be a key event in later periventricular white matter damage following IVH [20] and both systemic [24] and intraventricular [25] inflammation has been shown to alter the BBB permeability. Thus, characterizing the effects on and response of the choroid plexus following IVH is central in order to further understand the cellular and molecular mechanisms causing brain injury and leading to PHVD.

Following preterm IVH there is a release of red blood cells into the intraventricular space, with a subsequent hemolysis and release of cell-free Hb. We have previously shown that the oxidized form of extracellular Hb, metHb, is accumulated in the intraventricular CSF and is accompanied by increased levels of TNFα in CSF and in periventricular brain tissue [9]. In the present study, IVH was shown to be followed by an up-regulation of genes related to activation of TLR-4, IL1R1 and FAS pathways with induction of the transcription factor NF-Κβ in the choroid plexus. Activation of these cellular pathways was associated with an mRNA up-regulation of the chemotactic and pro-inflammatory effector molecules MCP-1, IL-6, IL-8, IL-1β and TNFα. A previous study reported activation of NF-Κβ in the choroid plexus and in the ependymal lining followed by functional barrier impairment in an adult rat model of IVH [25]. Further, conditional activation of NF-Κβ in the postnatal mouse has been associated with impaired ependymal ciliogenesis and development of hydrocephalus [26]. Indeed, we observed signs of severe choroid plexus epithelial damage, including mitochondrial swelling, caspase-3 activation and severe ciliar destruction at 72 hours following IVH.

Choroid plexus tissue sampled *in vivo* following IVH will contain a variable number of cell types, including plexus epithelium, capillary endothelium and macrophages. In order to further characterize the cellular and molecular events following IVH, we modeled the specific response of choroid plexus epithelium to IVH by exposing primary human choroid plexus epithelial cells to increasing concentrations of hemorrhagic CSF from preterm human infants with IVH. We found a clear dose-related up-regulation of the transcription factor NF-Κβ and effector molecules with a chemotactic function, MCP-1, IL-8, IL-6 and IL-1β. We did not observe significant changes in TNFα corresponding to those observed in the choroid plexus *in vivo* which suggests that this cytokine may be primarily generated by resident or invading immune cells. As described previously, following IVH and subsequent hemolysis of red blood cells, extracellular Hb-metabolites (which are oxy-, met-, ferryl Hb, free heme and reactive oxygen species (ROS)) are formed, released and accumulated in the intraventricular space. The previous study suggested that the oxidation of extracellular oxyHb to metHb is a critical event leading to inflammatory response in the CSF following IVH [9]. Here, we show that exposure of choroid plexus epithelial cells to metHb and heme, in similar concentrations to those detected in hemorrhagic CSF, induced a very similar pattern of cellular activation, inflammatory response and HO-1 expression as that observed by exposing cells to CSF from preterm human infants with IVH. This supports that extracellular Hb-metabolites are essential inducers of the responses observed *in vivo* following IVH. Furthermore, heme exhibited potent cytotoxic effect on plexus epithelial cells *in vitro*, whereas exposure to metHb was less cytotoxic but induced a clear activation of caspase-3/7, indicating that mechanisms induced by metHb result in apoptosis.

To further establish the causality between exposure to Hb-metabolites and the observed cellular and inflammatory response, *in vitro* choroid plexus epithelial cells were co-incubated with Hp during exposure to hemorrhagic CSF or metHb. Hp is an acute phase glycoprotein, produced mainly in the liver, which tightly binds Hb forming inert Hb-Hp complexes [27-31]. In the human body the predominant pathway for Hb clearance is the CD163-Hp-Hb system [27]. Co-incubation of plexus epithelial cells during exposure to metHb with equimolar concentrations of Hp resulted in a highly significant reduction of cytotoxicity and total inhibition of apoptosis as determined by caspase-3/7 activation. Furthermore, addition of Hp significantly reduced the mRNA up-regulation of genes involved in cellular activation, inflammatory response and HO-1 expression.

We further evaluated the reversing and protective effects of Hp *in vivo* by intraventricular administration in rabbit pups following detection of IVH. Analysis of structural integrity, using EM-IHC, displayed a preserved structure following intraventricular Hp injection. Furthermore, Hp administration significantly reduced cellular activation, inflammatory response and HO-1 expression at 24 hours, suggesting that administration of Hp blocks the toxic reactions of extracellular Hb-metabolites. The effects of systemic administration of Hp in conditions with increased intravasal levels of extracellular Hb has been well characterized. The resulting Hb-Hp complex impairs filtration and clearance of Hb dimers by the kidney, and directs Hb to CD163 on macrophages for a process of endocytosis and final degradation [32]. Within macrophages, the heme group of Hb is further degraded by the intracellular enzyme heme oxygenase (HO) [30] into bilirubin and CO, which both have been shown to have antioxidant and vasodilatory benefits. Therefore, the clearance of Hb offered by exogenous Hp indirectly protects against heme-mediated oxidative damage as well as other oxidative enzymatic reactions of Hb, and thus Hp can be viewed as a part of a human antioxidation mechanism [33]. The mechanisms whereby administrated Hp offers protection within the intraventricular space are not known, but are likely to be similar to those observed after systemic Hp administration, that is binding of Hb and subsequent clearance via CD163 positive macrophages. The effects of exogenous Hp on monocyte recruitment and CD163 expression warrant further investigation.

The endogenous concentration of Hp in the brain is very low and in a recent study in human adults the resting state capacity of the intrathecal CD163-Hp-Hb clearing system was described to be 50,000-fold lower than that of the circulation and this system was quickly saturated following subarachnoidal hemorrhage with a residual inability to deal effectively with extracellular Hb [34]. In addition, the normal circulating levels of Hp are reported to be very low in preterm infants, indicating a heightened vulnerability to extracellular Hb in this patient population [35,36]. To date, the concentration of intrathecal Hp in preterm infants has not been determined, but it is reasonable to assume that it is extremely low because systemic Hp contributes to intrathecal levels.

The present study has characterized the inflammatory response and structural damage in the choroid plexus following exposure to extracellular Hb-metabolites in the context of preterm IVH. The functional deficits observed in children following preterm IVH, mainly cerebral palsy and cognitive impairment, are probably primarily related to periventricular white matter damage including damage to subventricular neuronal progenitor cells. Continued study will therefore focus on evaluating the relative importance of extracellular Hb as a determinant of oligodendroglial functional impairment and neuronal death following preterm IVH. The ultimate aim will be to evaluate possible beneficial effects of scavenging extracellular Hb on long-term neurological function following IVH.

## Conclusion

The present data show that following IVH extracellular Hb is a causal inducer of inflammation, apoptosis and cytotoxicity in the immature brain.

New treatments for post-hemorrhagic brain damage and PHVD are sorely needed, as treatment strategies have not changed during the last 50 years. Emerging evidence shows that damage to the ependymal cells of the BBB is a key event in post hemorrhagic brain damage. Our data suggest that there is indeed a window of opportunity where targeting Hb-metabolites prior to substantial degradation could be a feasible approach. Treatment aiming at reducing Hb/metHb formation and inflammation in the intraventricular space, thus reducing damage to the ependymal cells lining the choroid plexus and the ventricles, might decrease the ensuing brain damage as well as the risk of developing hydrocephalus.

## Abbreviations

ANOVA: analysis of variance
BBB: blood-brain barrier
BSA: bovine serum albumin
CSF: cerebrospinal fluid
DMSO: dimethyl sulfoxide
EM-IHC: electron microscopy immunostaining
Fe^3+^: ferric
Fe^2+^: ferrous
GAPDH: glyceraldehyde-3-phosphate dehydrogenase
H&E: hematoxylin and eosin
Hb: hemoglobin
HCPEpiC: human primary choroid plexus epithelial cells
HO: heme oxygenase
Hp: haptoglobin
ICH: intracranial hemorrhage
IHC: immunohistochemistry
IL: interleukin
ip: intraperitoneal
iv: intravenous
IVH: intraventricular hemorrhage
LDH: lactate dehydrogenase
NF: nuclear factor
OD: optical density
PCR: polymerase chain reaction
PHVD: post-hemorrhagic ventricular dilatation
ROS: reactive oxygen species
TEM: transmission electron microscopy
TNF: tumor necrosis factor
